# Supplementary material for: Bacterial, Archaea, and Viral Transcripts (BAVT) Expression in Gynecological Cancers and Correlation with Regulatory Regions of the Genome
Source: Cancers (Basel). 2021 Mar 5;13(5):1109. doi: 10.3390/cancers13051109 (PMC7961894; doi:10.3390/cancers13051109)
Supplement: Supplementary file 1 [file cancers-13-01109-s001.pdf]

cancers

Supplementary Materials

Bacterial, Archaea, and Viral Transcripts (BAVT) Expression in Gynecological Cancers and Correlation with Regulatory Regions of the Genome

Jesus Gonzalez-Bosquet, Silvana Pedra-Nobre, Eric J. Devor, Kristina W. Thiel, Michael J. Goodheart, David P. Bender and Kimberly K. Leslie

A.

Name	Number of raw reads	Classified reads	Chordate reads	Microbial reads	Archaea reads	Viral reads
Tube1	47,405,479	100%	92.4%	0.871%	0.0281%	0.828%
Tube11	31,648,107	100%	94.1%	0.722%	0.0469%	0.662%
Tube13	28,679,244	100%	94.8%	1.38%	0.069%	1.29%
Tube15	22,557,926	100%	94.1%	0.895%	0.0558%	0.823%
Tube17	24,350,478	100%	90.7%	1.82%	0.0366%	1.77%
Tube18	36,677,140	100%	95.3%	0.752%	0.0452%	0.693%
Tube2	31,836,254	100%	93.3%	1.85%	0.0308%	1.8%
Tube3	24,368,492	100%	95.9%	0.749%	0.104%	0.631%
Tube4	30,703,146	100%	94.9%	1.65%	0.0395%	1.59%
Tube6	30,955,743	100%	93.3%	2.18%	0.0383%	2.13%
Tube8	26,835,434	100%	92.9%	1.97%	0.0622%	1.9%
Tube9	28,999,794	100%	92.1%	1.78%	0.0515%	1.72%

B.

Name	Number of raw reads	Classified reads	Chordate reads	Microbial reads	Archaea reads	Viral reads
Patient1	29,785,008	100%	93.9%	0.447%	0.0357%	0.403%
Patient10	36,469,173	100%	94.7%	0.647%	0.0286%	0.601%
Patient11	29,298,620	100%	95.4%	1.38%	0.051%	1.32%
Patient12	34,125,387	100%	95%	1.31%	0.0438%	1.25%
Patient13	25,969,661	100%	91.7%	2.49%	0.0315%	2.45%
Patient14	31,629,474	100%	91.4%	0.811%	0.0366%	0.762%
Patient15	22,319,317	100%	92.7%	1.84%	0.0721%	1.76%
Patient16	32,901,087	100%	95%	0.367%	0.0472%	0.308%
Patient17	32,134,740	100%	96.1%	0.458%	0.0499%	0.396%
Patient18	30,914,680	100%	92.3%	2.11%	0.0779%	2.01%
Patient19	23,671,248	100%	89.9%	2.83%	1.44%	1.31%
Patient2	29,830,848	100%	94.9%	1.04%	0.027%	0.993%
Patient20	43,015,725	100%	94.2%	1.94%	0.0583%	1.87%
Patient21	39,939,950	100%	92.2%	1.14%	0.0682%	1.06%
Patient22	28,661,428	100%	89%	2.34%	0.0594%	2.24%

Figure S1. Transcript expression of bacterial, viral and archaea organisms between normal tube tissue and ovarian cancer: (**A**). Number of aligned transcripts in normal tube and percentage of human (chordate) and non-human, including bacterial (or microbial), viral and archaea, among the total aligned RNA. (**B**). Number of aligned transcripts in 15 of the HGSC samples and percentage of human (chordate) and non-human, including bacterial (or microbial), viral and archaea, among the total aligned RNA.

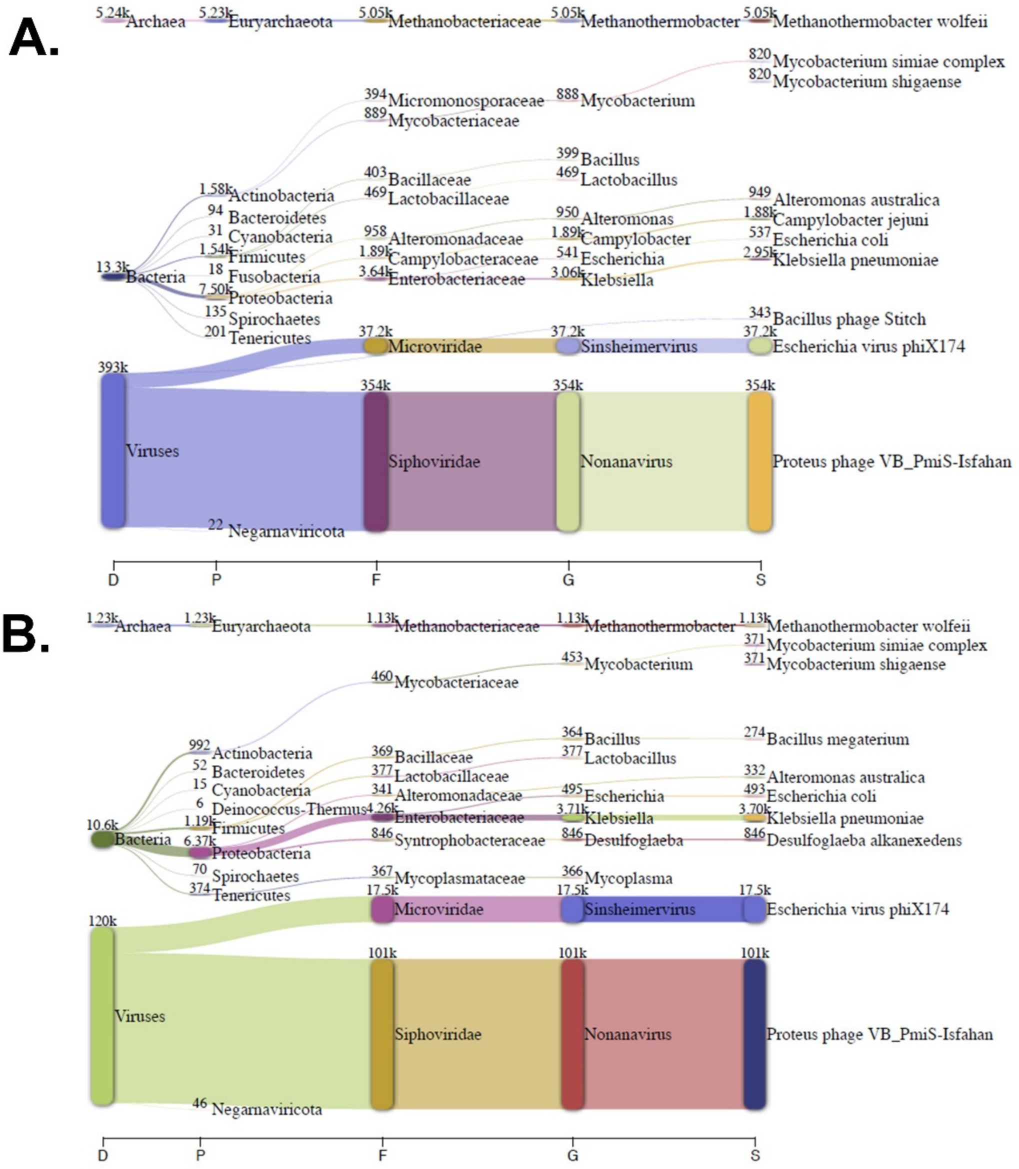

Figure S2. Sankey diagram visualization of the flow of values of human, bacterial, viral and archaea transcripts: the width of the diagram measures the size of transcripts. The differential expression analysis was performed at the Species Taxonomy rank; Taxonomy ranks: D: Domain; P: Phyla; F: Family; G: Genus; S: Species. (**A**). Normal tube sample. (**B**). HGSC sample.

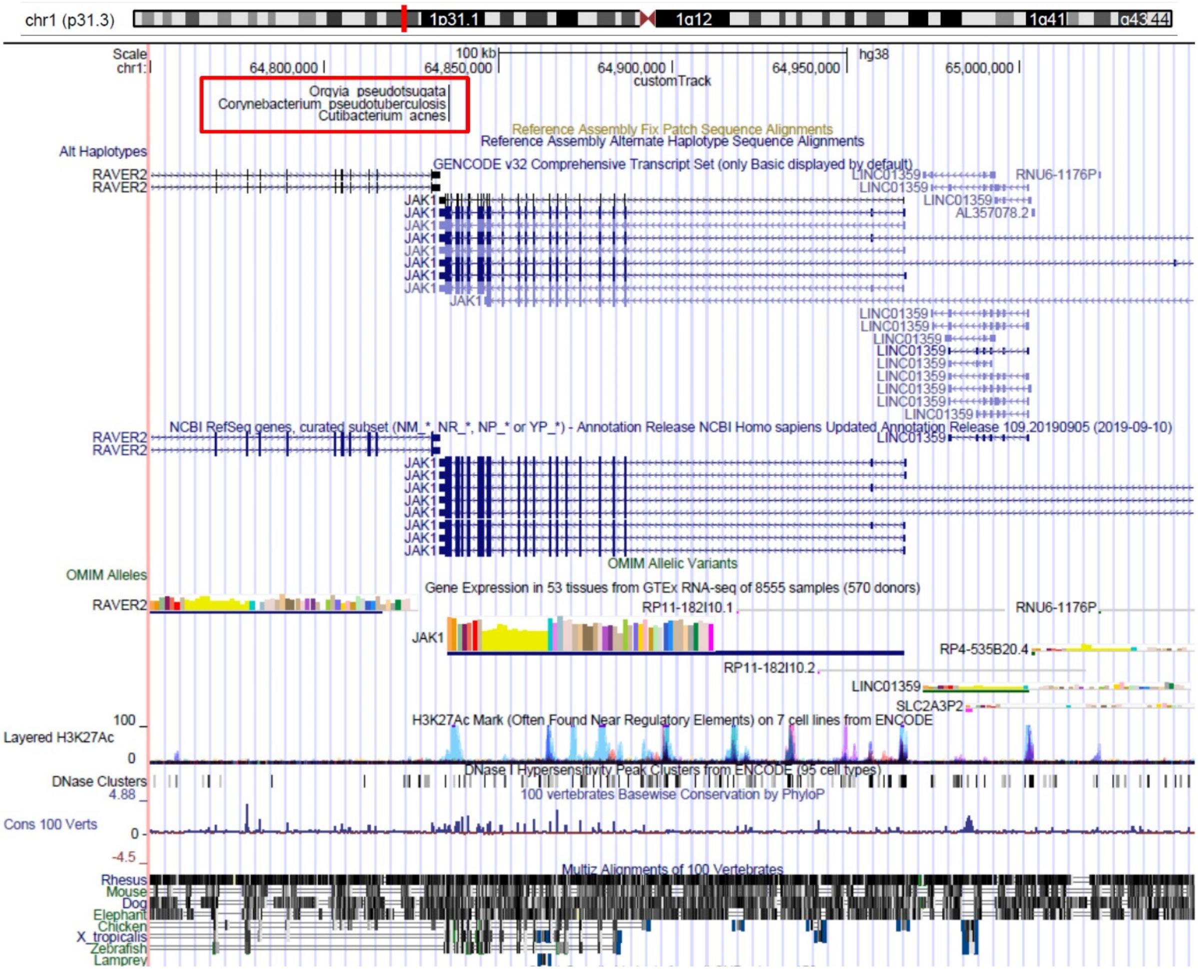

Figure S3. Sequence of methods with Cutibacterium Acnes (nucleotide NZ_AP019664.1) as an example. Best concordant aligned pairs were mapped with UCSC Genome Browser into the human genome (hg38): at the chromosomal marker (upper part of the panel, red rectangle) transcripts from Orgyia pseudotsugata, Corynebacterium pseudotuberculosis and Cutibacterium acnes were mapped at the 3′ region of the JAK1 gene (Janus kinase 1).

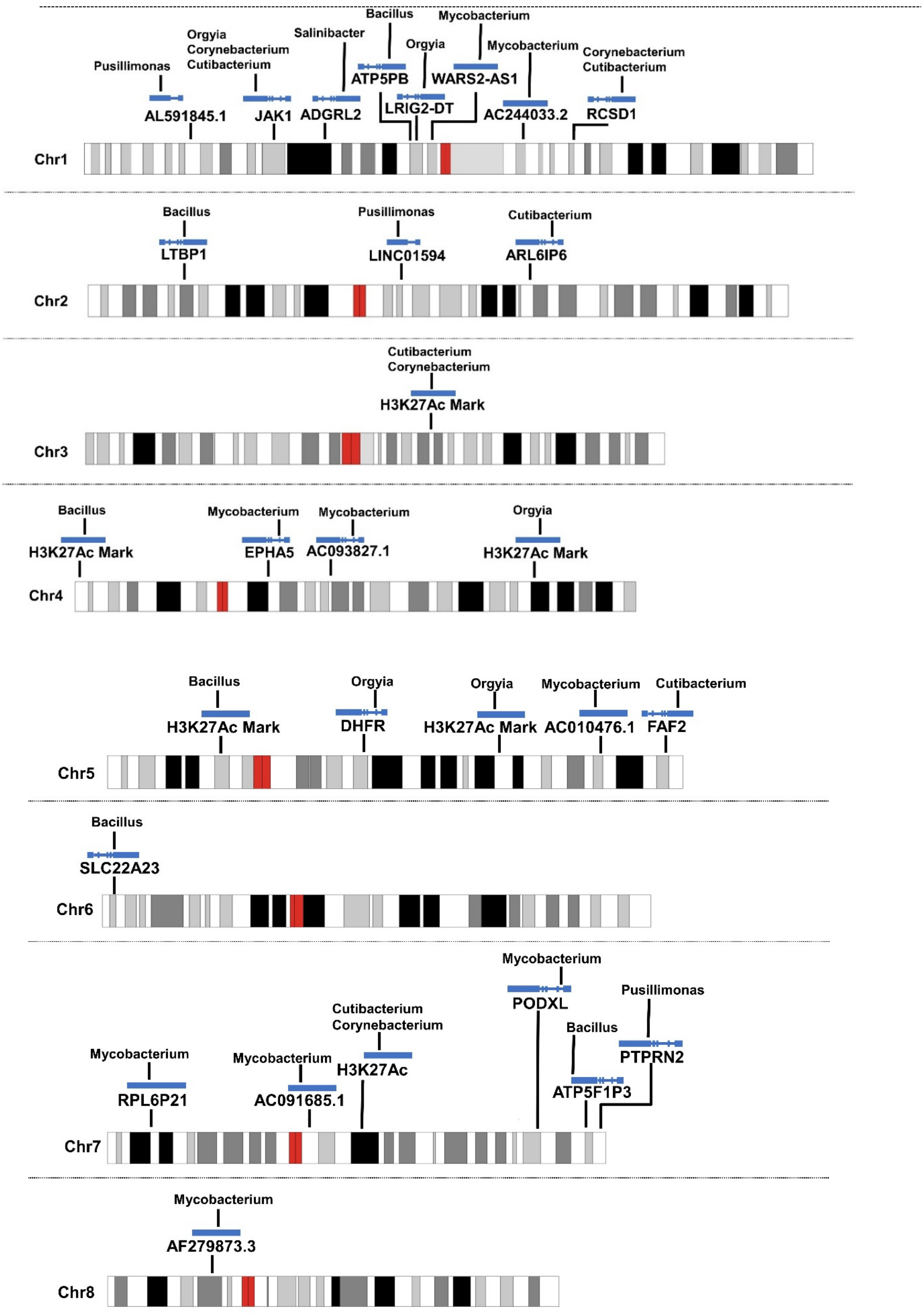

Figure S4. *Cont.*

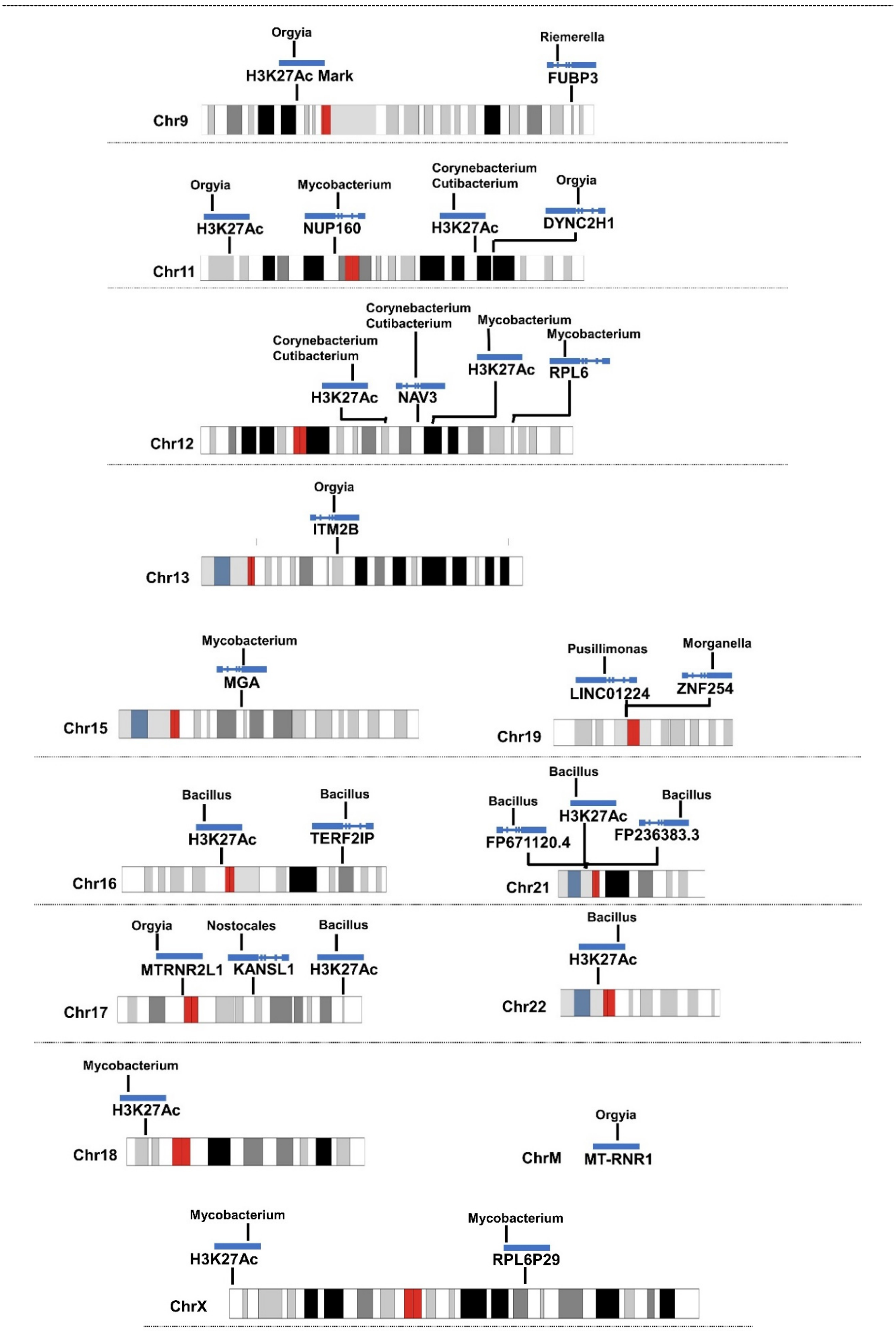

Figure S4. Detailed mapping of significant BAVT integrated into the human genome. Locations are provided in the 23-human karyotype view of the hg38 human genome assembly.

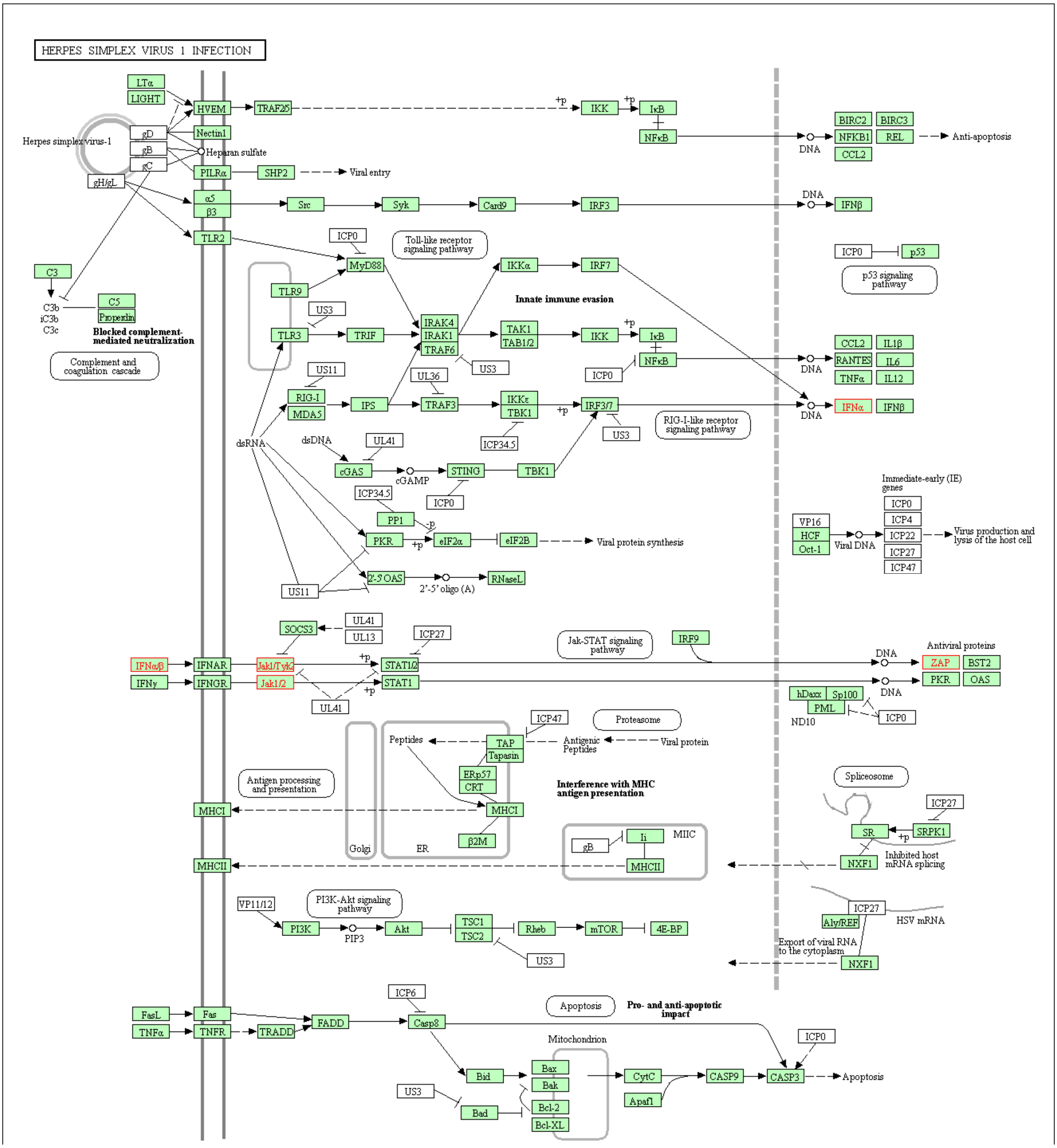

Figure S5. Herpes simplex virus 1 infection pathway (KEGG ID hsa05168). Highlighted in red genes introduced in the enrichment pathway analysis.

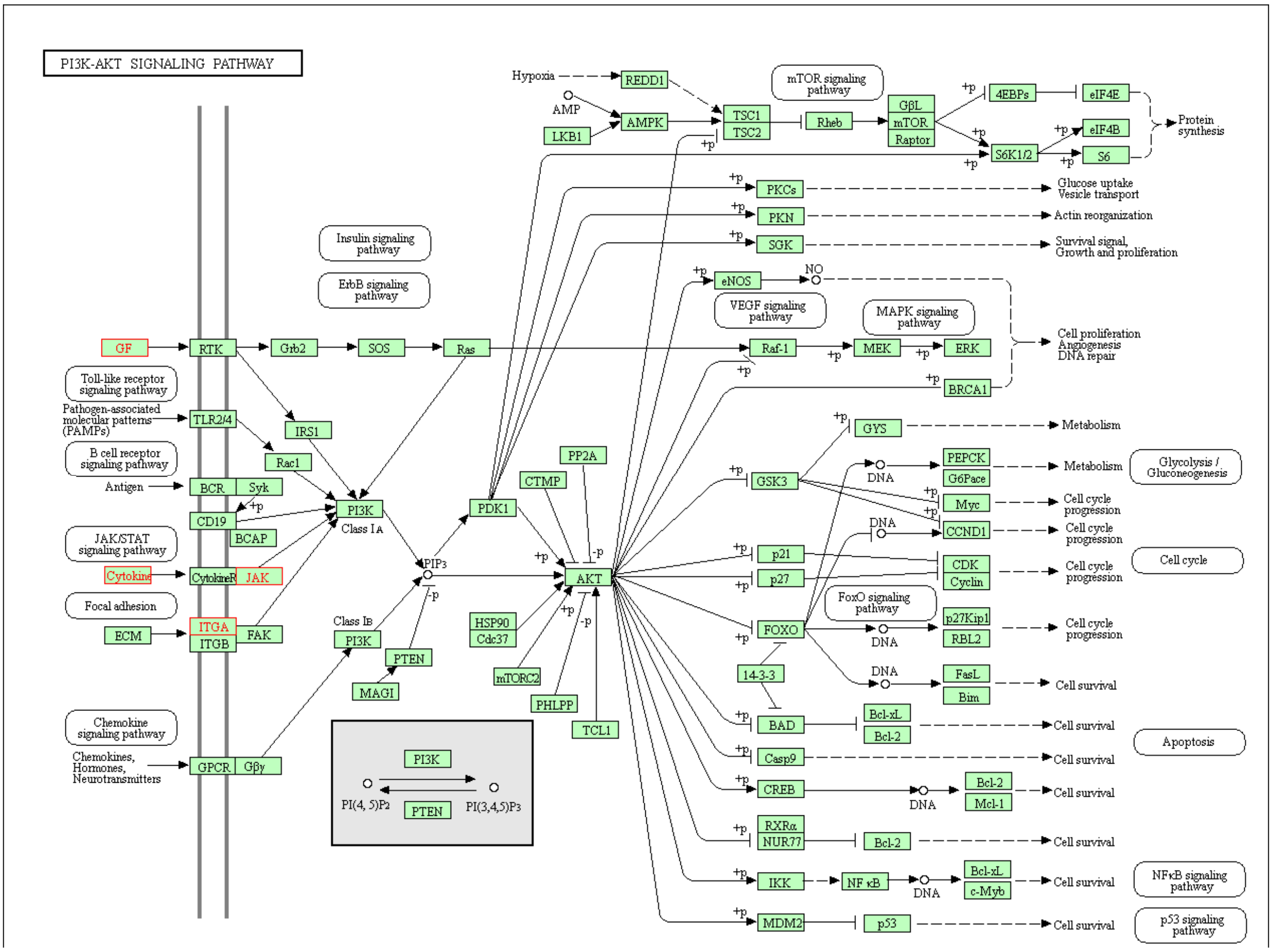

Figure S6. PI3K-Akt signaling pathway (KEGG ID hsa04151). Highlighted in red genes introduced in the enrichment pathway analysis.

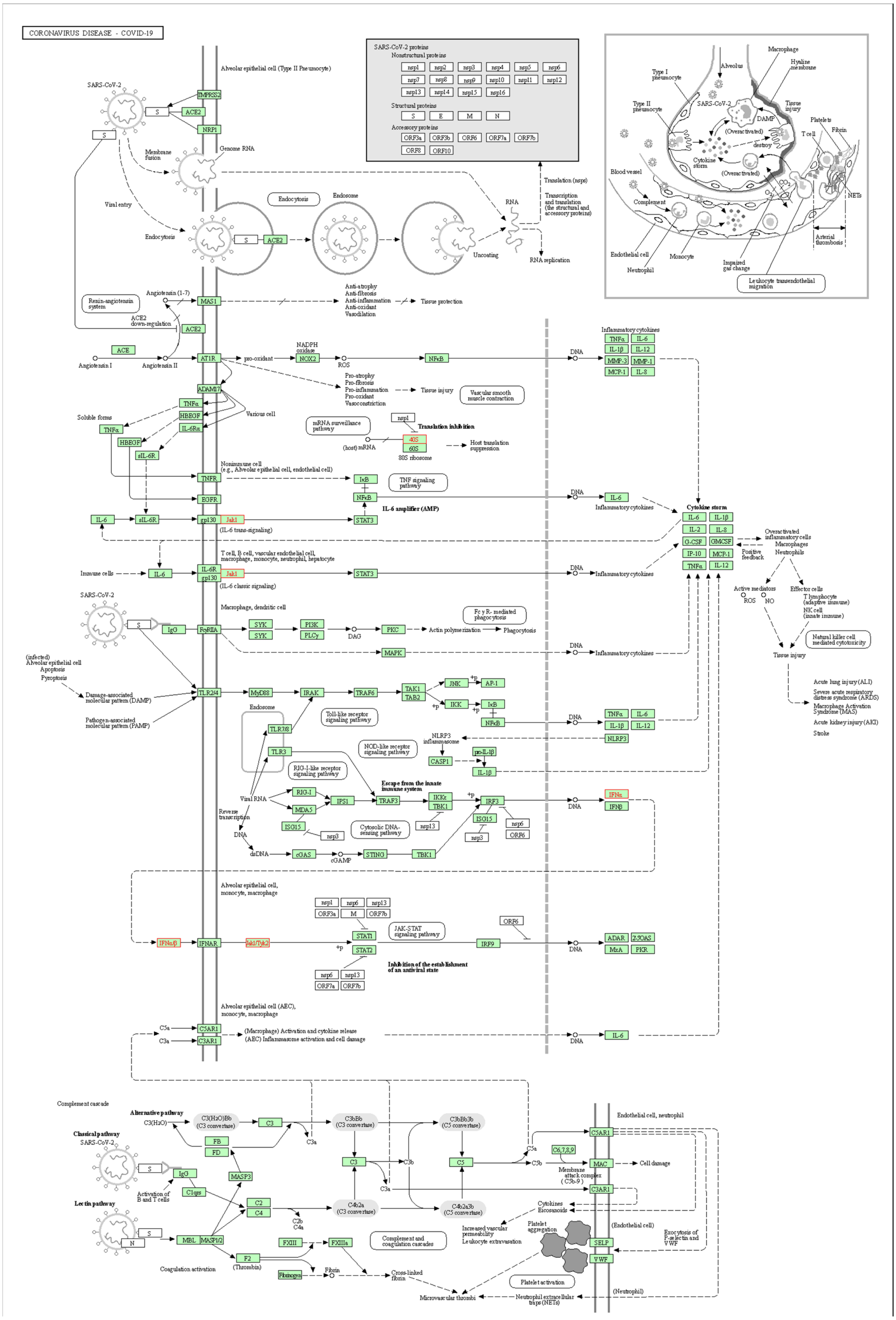

Figure S7. Coronavirus disease - COVID-19 pathway (KEGG ID hsa05171). Highlighted in red genes introduced in the enrichment pathway analysis.

Table S1. Methodological process using Cutibacterium Acnes (nucleotide NZ_AP019664.1) as an example. Best concordant aligned pairs were mapped with UCSC Genome Browser into the human genome (hg38). Genes and transcripts included in the same UCSC visualizing window that the mapped sequences were considered to be in the vicinity (around 25 kb per side).

Loci	Name	Transcripts Included	Name	Loci	Close Transcripts	Loci
chr1:36330639-36330670	Pusillimonas_sp	AL591845.1	LncRNA	chr1:36329630-36335406		
chr1:64835852-64835881	Orgyia_pseudotsugata	JAK1	Janus kinase 1	chr1:64833229-64966549		
chr1:64835852-64835881	Corynebacterium_pseudotuberculosis					
chr1:64835852-64835881	Cutibacterium_acnes					
chr1:81960259-81960362	Salinibacter_ruber	ADGRL2	Cell membrane; Multi-pass membrane protein	chr1:81836985-81991145		
chr1:111449478-111449581	Bacillus_licheniformis	ATP5PB	ATP synthase peripheral stalk-membrane subunit b	chr1:111449464-111462773		
chr1:111449832-111449874	Bacillus_licheniformis					
chr1:113067311-113067333	Orgyia_pseudotsugata	LRIG2-DT	LRIG2 divergent transcript	chr1:113011687-113073113		
chr1:119219359-119219508	Mycobacterium_shigaense	WARS2-AS1	LncRNA	chr1:119140391-119238057		
chr1:150054451-150054520	Mycobacterium_shigaense	AC244033.2	LncRNA	chr1:150045660-150067701		
chr1:167655490-167655514	Corynebacterium_pseudotuberculosis	RCSD1	RCSD domain containing 1	chr1:167630093-167706248		
chr1:167655490-167655514	Cutibacterium_acnes					
chr2:33063225-33063244	Bacillus_megaterium	LTBP1	latent transforming growth factor beta binding protein 1	chr2:32946953-33399509		
chr2:108177575-108177603	Pusillimonas_sp	LINC01594	long intergenic non-protein coding RNA 1594	chr2:108167126-108217886		
chr2:152742628-152742665	Corynebacterium_pseudotuberculosis	ARL6IP6	ADP ribosylation factor like GTPase 6 interacting protein 6	chr2:152718625-152759840		
chr2:152742628-152742665	Cutibacterium_acnes					
chr3:117799077-117799101	Corynebacterium_pseudotuberculosis	H3K27Ac Mark	Often Found Near Regulatory Elements	chr3:117798752-117799426		
chr3:117799077-117799101	Cutibacterium_acnes					
					AC092691.3	chr3:117790350-117794384
chr4:1618805-1618833	Bacillus_tropicus	H3K27Ac Mark	Often Found Near Regulatory Elements	chr4:1618689-1618949		
					FAM53A	chr4:1641293-1668741
chr4:65574052-65574201	Mycobacterium_shigaense	EPHA5	EPH receptor A5	chr4:65319563-65670343		
chr4:86870775-86870920	Mycobacterium_shigaense	AC093827.1	ribosomal protein L6 (RPL6) pseudogene	chr4:86870629-86871066		
chr4:155465824-155465974	Orgyia_pseudotsugata	H3K27Ac Mark	Often Found Near Regulatory Elements	chr4:155465673-155466125		
					AC097526.1	chr4:155442650-155448610
					MTCYBP17	chr4:155451617-155452405
chr5:35835162-35835186	Bacillus_tropicus	H3K27Ac Mark	Often Found Near Regulatory Elements	chr5:35835137-35835211		
					AC137810.1	chr5:35678484-35827018
					IL7R	chr5:35856891-35879603
chr5:80651405-80651556	Orgyia_pseudotsugata	DHFR	dihydrofolate reductase	chr5:80626226-80654983		
chr5:80651480-80651556	Orgyia_pseudotsugata					

chr5:123761384-123761529	Orgyia_pseudotsugata	H3K27Ac Mark	Often Found Near Regulatory Elements	chr5:123761238-123761675		
					LINC01170	chr5:124059794-124438520
					CSNK1G3	chr5:123545415-123615455
chr5:155111327-155111439	Mycobacterium_shigaense	AC010476.1	ribosomal protein L6 (RPL6) pseudogene	chr5:155111184-155112324		
chr5:176489965-176489987	Corynebacterium_pseudotuberculosis	FAF2	Fas associated factor family member 2	chr5:176448385-176510074		
chr5:176489965-176489987	Cutibacterium_acnes					
chr6:3360536-3360555	Bacillus_megaterium	SLC22A23	solute carrier family 22 member 23	chr6:3268962-3457022		
chr7:14070660-14070804	Mycobacterium_shigaense	RPL6P21	ribosomal protein L6 pseudogene 21	chr7:14070535-14071380		
chr7:64141664-64141813	Mycobacterium_shigaense	AC091685.1	LncRNA	chr7:64140495-64150058		
chr7:81790582-81790604	Corynebacterium_pseudotuberculosis	H3K27Ac Mark	Often Found Near Regulatory Elements	chr7:81790490-81790696		
chr7:81790582-81790604	Cutibacterium_acnes					
					HGF	chr7:81699010-81770047
chr7:137722577-137722726	Mycobacterium_shigaense	DGKI	diacylglycerol kinase iota	chr7:137381037-137846536		
chr7:152748905-152749052	Bacillus_licheniformis	H3K27Ac Mark	Often Found Near Regulatory Elements	chr7:152748313-152749644		
		ATP5F1P3	ATP synthase peripheral stalk-membrane subunit b pseudogene 3	chr7:152749079-152749735		
chr7:157813025-157813058	Pusillimonas_sp	PTPRN2	protein tyrosine phosphatase receptor type N2	chr7:157539056-158587773		
chr7:157813025-157813058	Pusillimonas_sp					
chr8:33859485-33859633	Mycobacterium_shigaense	AF279873.3	LncRNA	chr8:33604856-34038485		
chr9:33657197-33657345	Orgyia_pseudotsugata	H3K27Ac Mark	Often Found Near Regulatory Elements	chr9:33656601-33657941		
					PTENP1 antisense RNA	chr9:33676785-33687990
chr9:130582985-130583136	Riemerella_anatipestifer	FUBP3	far upstream element binding protein 3	chr9:130579577-130638352		
chr11:10509546-10509697	Orgyia_pseudotsugata	H3K27Ac Mark	Often Found Near Regulatory Elements	chr11:10509394-10509849		
chr11:10509621-10509697	Orgyia_pseudotsugata					
					MTRNR2L8	chr11:10507894-10509186
					RNF141	chr11:10511673-10541187
chr11:47796168-47796251	Mycobacterium_shigaense	NUP160	nucleoporin 160	chr11:47778087-47848467		
chr11:97201026-97201048	Corynebacterium_pseudotuberculosis	H3K27Ac Mark	Often Found Near Regulatory Elements	chr11:97200934-97201140		
chr11:97201026-97201048	Cutibacterium_acnes					
					LINC02553	chr11:97222644-97259987
chr11:103410399-103410548	Orgyia_pseudotsugata	DYNC2H1	dynein cytoplasmic 2 heavy chain 1	chr11:103109426-103479863		
chr12:66105265-66105298	Corynebacterium_pseudotuberculosis	H3K27Ac Mark	Often Found Near Regulatory Elements	chr12:66105129-66105434		
chr12:66105265-66105298	Cutibacterium_acnes					
					LLPH	chr12:66116555-66130750
chr12:77998906-77998932	Corynebacterium_pseudotuberculosis	NAV3	neuron navigator 3	chr12:77830894-78213010		
chr12:77998906-77998932	Cutibacterium_acnes					

chr12:83151917-83152057	Mycobacterium_shigaense	H3K27Ac Mark	Often Found Near Regulatory Elements	chr12:83151353-83152621		
					RPL6P25	chr12:83151331-83152190
					TMTC2	chr12:82686906-83134866
					AC090680.1	chr12:83171590-83172740
chr12:112405350-112405975	Mycobacterium_shigaense	RPL6	ribosomal protein L6	chr12:112405199-112409641		
chr12:112405350-112405378	Mycobacterium_shigaense					
chr12:112405852-112405977	Mycobacterium_shigaense					
chr13:48240848-48240977	Orgyia_pseudotsugata	ITM2B	integral membrane protein 2B	chr13:48232612-48261875		
chr13:109424124-109424200	Orgyia_pseudotsugata	H3K27Ac Mark	Often Found Near Regulatory Elements	chr13:109423817-109424509		
					AL163541.1	chr13:109478739-109731700
					LINC00399	chr13:109400696-109401641
chr15:41718496-41718642	Mycobacterium_shigaense	MGA	MAX dimerization protein MGA	chr15:41660412-41769943		
chr16:34160641-34160661	Bacillus_megaterium	H3K27Ac Mark	Often Found Near Regulatory Elements	chr16:34160557-34160745		
					LINC00273	chr16:34158585-34160036
chr16:75709727-75709856	Bacillus_licheniformis	TERF2IP	TERF2 interacting protein	chr16:75647791-75761830		
chr17:22522619-22522767	Orgyia_pseudotsugata	MTRNR2L1	MT-RNR2 like 1	chr17:22523111-22524663		
chr17:46034831-46034982	Nostocales_cyanobacterium	KANSL1	KAT8 regulatory NSL complex subunit 1	chr17:46029916-46193198		
chr17:77234127-77234154	Bacillus_tropicus	H3K27Ac Mark	Often Found Near Regulatory Elements	chr17:77234015-77234266		
					SEC14L1	chr17:77140932-77214329
					SEPTIN9-DT	chr17:77257737-77274326
chr18:6462270-6462298	Mycobacterium_shigaense	H3K27Ac Mark	Often Found Near Regulatory Elements	chr18:6462154-6462414		
					RNU6-349P	chr18:6454962-6455068
chr19:23406864-23406886	Pusillimonas_sp	LINC01224	LncRNA	chr19:23399470-23408091		
chr19:24103242-24103323	Morganella_morganii	ZNF254	zinc finger protein 254	chr19:24033405-24129961		
chr21:8210240-8210260	Bacillus_megaterium	FP671120.4	LncRNA	chr21:8197620-8227646		
chr21:8254448-8254468	Bacillus_megaterium	H3K27Ac Mark	Often Found Near Regulatory Elements	chr21:8254364-8254552		
					FP671120.7	chr21:8254592-8255514
chr21:8393275-8393295	Bacillus_megaterium	FP236383.3	LncRNA	chr21:8380643-8410645		
chr21:8437485-8437505	Bacillus_megaterium	FP236383.3	LncRNA	chr21:8380665-8454792		
chr22_KI270733v1_random:126540-126560	Bacillus_megaterium	RNA45SN4	RNA, 45S pre-ribosomal N4	chr22_KI270733v1_random:126267-126833		
chr22_KI270733v1_random:171621-171641	Bacillus_megaterium	H3K27Ac Mark	Often Found Near Regulatory Elements	chr22_KI270733v1_random:171537-171725		
					5_8S_rRNA	chr22_KI270733v1_random:173956-174108
					AL353644.2	chr22_KI270733v1_random:167218-167307

chr22:11630156-11630176	Bacillus_megaterium	H3K27Ac Mark	Often Found Near Regulatory Elements	chr22:11630072-11630260		
					CT867976.1	chr22:11474744-11479643
					AC138776.1	chr22:11827523-11910358
chrM:1161-1237	Orgyia_pseudotsugata	MT-RNR1	mitochondrially encoded 12S ribosomal RNA	chrM:648-1601		
chrUn_GL000220v1:109687-109707	Bacillus_megaterium	LOC100507412	uncharacterized LOC100507412	chrUn_GL000220v1:97129-126696		
chrUn_GL000220v1:153659-153679	Bacillus_megaterium	H3K27Ac Mark	Often Found Near Regulatory Elements	chrUn_GL000220v1:153575-153763		
					LOC100507412	chrUn_GL000220v1:97129-126696
chrX:1174838-1174984	Mycobacterium_shigaense	H3K27Ac Mark	Often Found Near Regulatory Elements	chrX:1174250-1175572		
					CRLF2	chrX:1187549-121263
chrX:98252268-98252417	Mycobacterium_shigaense	RPL6P29	ribosomal protein L6 pseudogene 29	chrX:98251679-98252543		
